# Atherosclerosis in HIV Patients: What Do We Know so Far?

**DOI:** 10.3390/ijms23052504

**Published:** 2022-02-24

**Authors:** Anastasia V. Poznyak, Evgeny E. Bezsonov, Evgeny E. Borisov, Andrey V. Grechko, Andrey G. Kartuesov, Alexander N. Orekhov

**Affiliations:** 1 Institute for Atherosclerosis Research, Skolkovo Innovative Center, 121609 Moscow, Russia; 2 AP Avtsyn Research Institute of Human Morphology, 3 Tsyurupa Street, 117418 Moscow, Russia; evgeny.bezsonov@gmail.com (E.E.B.); borisovevgenij5@gmail.com (E.E.B.); 3 Laboratory of Angiopathology, Institute of General Pathology and Pathophysiology, 8 Baltiiskaya Street, 125315 Moscow, Russia; andkartuesv@gmail.com; 4 Department of Biology and General Genetics, I.M. Sechenov First Moscow State Medical University (Sechenov University), 8 Izmailovsky Boulevard, 105043 Moscow, Russia; 5 Federal Research and Clinical Center of Intensive Care Medicine and Rehabilitology, 14-3 Solyanka Street, 109240 Moscow, Russia; avg-2007@yandex.ru

**Keywords:** atherosclerosis, HIV, immunity

## Abstract

For the past several decades, humanity has been dealing with HIV. This disease is one of the biggest global health problems. Fortunately, modern antiretroviral therapy allows patients to manage the disease, improving their quality of life and their life expectancy. In addition, the use of these drugs makes it possible to reduce the risk of transmission of the virus to almost zero. Atherosclerosis is another serious pathology that leads to severe health problems, including disability and, often, the death of the patient. An effective treatment for atherosclerosis has not yet been developed. Both types of immune response, innate and adaptive, are important components of the pathogenesis of this disease. In this regard, the peculiarities of the development of atherosclerosis in HIV carriers are of particular scientific interest. In this review, we have tried to summarize the data on atherosclerosis and its development in HIV carriers. We also looked at the classic therapeutic methods and their features concerning the concomitant diagnosis.

## 1. Atherosclerosis

Atherosclerosis is a chronic inflammatory disease of the arterial wall, which quite often results in invalidity or fatal events. Typical signs of the final stage of atherosclerosis are the defeat of the intimate layer of the arterial wall and plaque storage [1,2]. The erosion or breaking of atherosclerotic plaques leads to thrombotic events that can theoretically lead to death. Years of active research demonstrate that atherosclerosis is characterized by a tricky pathogenesis, the main aspects of which are lipid storage and chronic inflammation in the arterial wall. As a rule, atherosclerosis is linked with changes in lipid metabolism and hypercholesterolemia [3]. A high circulating modified low-density lipoproteins level (LDL) is known as a reason for cardiovascular disease (CVD) development. The pathogenesis of the disease is much more complicated than changes in lipid metabolism and includes many factors, of which the key factor is inflammation [4].

It is believed that the sequence of pathological events resulting in atherosclerosis progression is provided by local endothelial dysfunction due to factors such as turbulence of blood flow near the places of curves and/or bifurcations of the arteries. The endothelium of blood vessels reacts to mechanical stress by activation, which then results in the recruitment of circulating immune cells [5]. Circulating monocytes ‘stick’ to the affected section of the arterial wall and get inside, differentiating into macrophages that intensively participate in the lipid devourment through phagocytosis and generate the foam cells enriching atherosclerotic plaques [6,7].

A comprehensive study of the formation of atherosclerotic lesions is aggravated because this process in humans and possible model animals can be radically different. Yet, the key contours of this process may be determined. “Fatty streak” is the so-called initial atherosclerotic lesion stage. The “fatty streak” is a vascular wall’s zone that is distinguished by the intracellular storage of lipids by foam cells, which in turn also contain vascular smooth muscle cells (VSMCs) and T-lymphocytes. If chronic endothelial damage remains, “fatty streaks” are able to further progress to atherosclerotic lesions [8]. In developing lesions, the intracellular accumulation of lipids includes several types of cells. The recruited macrophages internalize LDL particles via phagocytosis and promote the development of local inflammation mediators. Often, resident intimate cells are strenuously involved in this process. Stellar-shaped microvessels create a three-dimensional cellular network in the subendothelial layer of the intima, generating exposure both with each other and endothelial cells, thus providing tissue homeostasis. Due to phenotypic changes in pericytes, this network is destroyed in atherosclerotic plaques, which results in the loss of intercellular contacts and in a growth in the production of extracellular matrix components. The vascular smooth muscle cells implicated in the pathological process are able to experience phenotypic switching, perhaps receiving proliferative and secretory properties [9,10].

During the late stages of atherosclerosis development, these cells obtain a stable fibrous membrane that divides them from the vessel milieu. The violation of the stability of the plaque happens due to the exhaustion and tear of the fibrous membrane, which is facilitated by matrix metalloproteinases (MMP) instigating the degradation of the extracellular matrix [11,12]. As well as other inflammatory cells, macrophages are an important resource of these enzymes in the plaque. Liable plaque erosion mechanisms require further study. These processes are particularly complicated to model in atherosclerotic animals. Inflammatory events, such as platelet-mediated local neutrophil enabling, myeloperoxidase release, toll-like receptor (TLR-2) signaling, and neutrophil-mediated damage, appear to be important in this process [13,14].

Due to atherosclerotic plaques, the lumen of the blood vessel narrows, resulting in both ischemia and metabolic changes in the alimented tissues. More dangerous is thrombogenesis, which occurs due to unstable plaques, and occasionally on the surface of intact plaques, which frequently leads to lethal consequences [15].

## 2. Cardiovascular Disease in HIV Patients

Cardiovascular disease among HIV carriers led to big changes with the advent of antiretroviral therapy (ART). Once the HIV epidemic began, the main variants of cardiovascular diseases that were directly associated with HIV were dilated cardiomyopathy, pericardial diseases, pulmonary hypertension, malignant neoplasms, as well as opportunistic infections. After HIV-infected patients started receiving antiretroviral therapy, they became increasingly at risk of more common cardiovascular complications linked with atherosclerosis, e.g., stroke, heart failure, and also myocardial infarction [16].

The implementation of antiretroviral therapy elevated the survival rate of HIV carriers to a level that is quite close to population survival rate. Despite the fact that a smaller number of HIV carriers die from AIDS-related complications, the occurrence of non-AIDS-related comorbidities, including CVD-related atherosclerosis, is still higher in contrast with the HIV-negative control [17,18]. According to statistics, in America, cardiovascular diseases are the second leading cause of non-AIDS-related mortality, while in Europe, cardiovascular diseases among HIV carriers are the third leading cause of mortality. It is known that HIV carriers have a higher tendency to traditional risk factors, including hypertension, diabetes, and dyslipidemia. Despite the increased control of these traditional factors, there is quite strong evidence that HIV carriers are still at a higher (about 1.5–3 times) risk of developing cardiovascular disease [19,20].

Two significant clinical trials have given useful information regarding ART and the timing of its use in HIV infection in connection with CVD: the Strategies for Conducting Antiretroviral Therapy (SMART) and Strategic Timing of Antiretroviral Treatment (START) studies [21]. The SMART study demonstrated that consecutive use of ART in individuals with a CD4^+^ cell count below 350/µL led to a reduction in AIDS-related side effects and cases of CVD. Those who postponed or interrupted treatment had a 70% higher risk of developing cardiovascular disease, which indicates the need for constant ART to prevent the chronic inflammation linked with HIV and lower the risk of CVD. In the START study, there was a 40% decrease in AIDS-related cases with immediate ART administration, though this did not prevent the development of CVD [22].

Despite the SMART study, as well as the START study, supporting the idea that antiretroviral therapy based on stricter CD4^+^ thresholds will probably lead to a decrease in the frequency of CVD, that is not enough to use ART to avoid the risk of cardiovascular events in HIV carriers [23]. The Veterans Aging Study cohort’s data make it clear that HIV-infected patients had an increased risk of acute myocardial infarction (risk factor 1.48), even after adjusting for Framingham risk factors, concomitant diseases, and drug use. Therefore, non-ART interventions are necessary to lower the chances of CVD events among the population with HIV carriers and improve immune function [24,25].

HIV-infected patients who do not have a viral load in the blood plasma without ART are known as “elite controllers”; they have high coronary artery atherosclerosis and elevated immune activation, including high levels of soluble CD163 in plasma (sCD163) [26,27]. In another cohort, HIV “elite controllers” had a higher average carotid artery intima thickness (CIMT) than in uninfected subjects, even after adjusting for traditional cardiovascular risk factors. These studies strengthen the role of inflammation as a key mediator of HIV-associated cardiovascular disease [28].

## 3. Underlying Cellular Mechanisms 

Everything that we currently know about HIV-related atherosclerosis has become known only through clinical studies and a small number of mechanical experimental studies. Thus, what we do know and comprehend about atherosclerosis in HIV is limited to the knowledge about traditional (non-HIV) atherosclerosis [29].

The main link in HIV-related atherosclerosis is probably focused on macrophages and their decisive role in the inflammatory process and plaque development. Despite the fact that T cells are a key cellular repository for HIV infection, monocytes and macrophages also keep HIV replication. Therefore, these cells continue to be permanently infected. In HIV carriers who are constantly receiving antiretroviral therapy, the infection persists, and the virus in T cells and monocytes/macrophages remains hidden, integrating with the host gene in order to reappear when antiretroviral therapy is interrupted or stops. In the latent phase, when the viral load is reduced and cannot be detected in human blood, powerful viral regulatory proteins (Tat, Nef, and others) are produced at low levels in both T cells and monocytes and are able to change their function [30,31,32].

As mentioned earlier, biomarker studies have given important information about the cellular mechanisms underlying HIV-related atherogenesis. We have summed up all the results of these studies concerning the correlation of cellular markers with the promotion of HIV-associated CVD. In these studies, we agree on the importance of the immune cell trigger and inflammation, primarily due to the monocyte/macrophage trigger, in the pathogenesis of HIV-related atherogenesis [33,34]. As a result of these studies, it became possible to clarify the hypothesis that the key underlying cellular mechanism seems to be chronic inflammation mediated by macrophages. While studying clinical biomarkers, a lack of attention was paid to smooth muscle cells (SMC), endothelial cells, or platelet activation, and few correlations were found with HIV-CVD. A number of studies involving large cohorts have found a significant difference between cardiovascular disease and the engagement of endothelial cells and platelets. It is highly important not to lose sight of their atherogenic role when elucidating mechanisms of these diseases [35,36].

Our awareness of the molecular mechanisms fundamental to HIV-related atherosclerosis is not as broad as our awareness of cellular mechanisms. Even in those HIV carriers who receive antiretroviral therapy, the interaction of HIV with the host immune cells and endothelial cells can enable several molecular events, including an elevation in oxidative stress, endoplasmic reticulum (ER) stress, the development of inflammatory cells, and a violation of the regulation of autophagy. These cellular mechanisms are of particular importance in traditional atherogenesis [37,38]. We are willing to concentrate on the potential role of these molecular mechanisms in the formation of HIV-related atherogenesis.

### 3.1. Oxidative Stress

Reactive oxygen species (ROS) are extremely active molecules formed due to redox reactions in cellular processes, such as oxidative metabolism and the degradation of macromolecules and their substrates, as well as protein folding in the ER. The reactive oxygen species’ main source are electron transport chains, nicotinamide adenine dinucleotide phosphate (NADPH) oxidase enzymes, or the cytochrome P450 system [39]. ROS are produced in excess on a regular basis. For this reason, eukaryotic cells have created mechanisms for neutralizing, restoring, and inhibiting their production. Many mechanisms are involved in the neutralization or removal of ROS, including low-molecular-weight antioxidants, e.g., glutathione, NADPH reductase enzymes, superoxide dismutase, and heme oxygenase. Yet, when the production of reactive oxygen species increases or the antioxidant capacity of the cell decreases, ROS is able to become pathological [40].

ROS plays an important role in the progression of atherosclerosis. NADPH oxidase accumulates quite strongly in phagocytic cells (macrophages are also participating), where they are extremely important for protecting the host from ingested pathogens. NADPH oxidase is also expressed in endothelial cells and SMCs. The atherogenic role of members of the NADPH oxidase enzyme family has been widely analyzed. In the course of the studies, mice with zero apolipoprotein E (*ApoE*^−/−^) were used, and this protein was found to play a decisive role both in the proliferation of endothelial cells and SMC and also in the migration of macrophages [41,42,43].

In vitro and in vivo models of HIV infection, as well as primary patient samples, demonstrate an elevated level of ROS and a low level of oxidative stress. ROS levels and their effects (e.g., oxLDL and oxidized nucleic acids) are increased in untreated patients in contrast to patients receiving ART [44,45]. The increase in ROS is the result of both production elevation and antioxidant capacity reduction. HIV carriers who have an increased oxidative stress due to a mutation that affects the antioxidant ability have a raised viral load in the HIV plasma, a reduction in the number of CD4^+^ T cells, and elevated cytotoxicity. Plenty of HIV proteins, including Nef, raise ROS production, which results in endothelial dysfunction [46]. Multiple studies have also demonstrated that specific ART regimens elevate ROS development by mediating endothelial dysfunction, for which mitochondrial-targeted antioxidants and antioxidant therapy are protective. These data prove that an elevation in chronic oxidative stress as a result of HIV infection or ART mediates endothelial dysfunction and elevations in the production of OXLPNP [47]. Nevertheless, the following question remains open and requires further research: Do the increased production of ROS and its intracellular effects, including stress, participate in the activation of HIV-associated immune cells and atherogenesis?

### 3.2. ER Stress

The ER is a site of synthesis and modification of lipids and proteins, as well as protein folding, maturation, and transportation of collected proteins. The ER is likewise involved in the regulation of calcium and intracellular redox potential. All these function disturbances result in a voltage ER stress, which enables a signaling cascade also known as the unfolded protein response (UPR). The unfolded protein response is perceived and triggered by three proteins: (1) inositol-requiring 1 (IRE1), (2) activating transcription factor 6 (AT6), and (3) protein kinase RNA-like ER kinase (PERK) [48]. Triggering these stress sensors leads to compensatory mechanisms for ER stress release, which also involves protein translation suppression, the induction of chaperone molecules, and the enhancement of ER biogenesis. Apoptosis can be the result of prolonged activation and ER stress or the induction of other cellular stress signaling pathways (e.g., oxidative stress) [49,50].

Studies were conducted to determine the significance of ER stress and ER-induced apoptosis. Since UPR is a trigger during the formation of all stages of atherosclerotic lesion, theoretically stress is able to precede several stages of atherosclerosis. The shear stress caused by blood flow leads to ER stress in endothelial cells and also to endothelial cell dysfunction [51]. Prolonged ER stress and apoptosis in SMCs is able to cause low collagen production, as well as the development of a fibrous cap, which eventually results in instability and plaque break. The ER is the site of cholesterol-induced cytotoxicity of macrophages, and ER stress is able to induce macrophage apoptosis [52]. OxLDL is able to induce ER stress in animal models of atherosclerosis. In mouse models of atherosclerosis, the suppression of ER stress or the deletion of ER-stress genes play a protective role of the cell against atherosclerosis. ER stress also manifests itself in progressive human plaques. ER stress: (1) elevates endothelial activation; (2) elevates foam cells development; (3) elevates apoptosis in macrophages participating in necrotic lipid nucleus development; and (4) lowers SMC proliferation and collagen generation, which leads to instability of the plaque [53,54]. 

Apoptosis of the vascular system can also be caused by the fact that an increase in ROS and the release of soluble cellular factors and host factors after HIV infection leads to the induction of a response to ER stress. In turn, HIV causes ER stress via interference with host genes, which results in ER calcium homeostasis instability. Some HIV proteins are able to cause an instability of calcium in endothelial cells and are also able to cause apoptosis as a result of stress, which, in turn, leads to endothelial dysfunction. Eventually, the HIV-mediated induction of apoptosis of human endothelial cells involves ER stress and mitochondrial dysfunction. The HIV envelope protein, gp120, induces type 1 programmed cell death as a result of ER stress using the IRE1a, JNK, and AP-1 pathways. HIV ART induces ER stress, elevates inflammatory cytokine synthesis, including tumor necrosis factor alpha (TNF-α) and IL-6, greatly elevates apoptosis, and contributes to foam cell development in macrophages. The integrase inhibitor raltegravir is able to eliminate the inflammatory response caused by an HIV protease suppressor and the foam cell development by suppressing ER-stress, presuming that the inclusion of raltegravir is able to lower the ER-stress-induced cardiovascular disease associated with current ART [55,56].

### 3.3. NLRP3 Inflammasome Activation

The onset of the inflammatory process is associated with a part of the immune system’s response with a high degree of preservation to malicious stimuli, including pathogenic microorganisms, such as HIV, cellular debris, and other stimuli. Therefore, the immune system triggers the promotion of inflammatory cytokines. The inflammasome hyperactivation promotes the development of autoimmune diseases and chronic inflammatory diseases, including atherosclerosis [57]. The inflammation development depends on the identification of pathogen-linked molecular patterns (PAMPs) or danger-related molecular patterns (DAMPs) by pattern recognition receptors (PRR), such as Toll-like receptors (TLR), NOD-like receptors (NLR), or absent in melanoma 2 (AIM)-like receptors (ALRs). In response to these signals, NLR or ALR oligomerize, forming an inflammatory complex. This inflammatory complex further recruits and enables zymogen pro-caspase-1, which activates pro-IL-1β and pro-IL-18, resulting in the formation of active cytokines, IL-1β and IL-18, and subsequent cascades of inflammation. IL-1β has a key role in stimulating inflammation by uniting with its receptor and inducing a greater number of pro-inflammatory cytokines, including TNF-α [58,59,60]. 

NLRP3 inflammation is triggered by hazard signals participating in atherogenesis, oxLDL, and cholesterol crystals. A fairly large number of both clinical and experimental data indicate that the formation of inflammasomes/activation of caspase-1 play a decisive role of atherosclerosis initiation and development, including progressive plaque breakage [61,62].

The enabled levels of caspase-1 increase intensively during early HIV infection. In patients with a high CD4 content, the level of caspase-1 decreased quite promptly, while in patients with a low CD4 content, the level of caspase-1 remained increased after 1 year of HIV infection. This process may be separate and unrelated to the viral envelope and may also be enhanced by concomitant diseases associated with HIV [63].

In addition, the NLRP3 inflammatory trigger in HIV-infected T cells contributes to the pathogenesis of depletion and T cell enablement in HIV carriers. Several studies have demonstrated that the formation of monocyte/macrophage inflammasomes is able to contribute directly to HIV-related atherogenesis. Within both infected immune and endothelial HIV infection, they are able to trigger dangerous signaling cascades that run inflammatory cell development [32]. HIV infection mediates the trigger of NLRP3 inflammation in human macrophages, which causes the release of bioactive IL-1β and IL-18 through TLR8-mediated mechanisms in vitro. IL-1β induces its own development and the synthesis and expression of other cytokines linked with HIV-CVD, such as IL-6. In HIV-infected rhesus macaques that were on a diet high in fat/cholesterol, the IL-18 levels in the blood plasma were noticeably correlated with the area of atherosclerotic plaques. In the plaques of these animals, IL-18 was specifically colocalized with CD68^+^ macrophages and not CD3^+^ T cells [64].

This outcome indicates that the role of the NLRP3 inflammatory trigger can vary and depends on which type of cells are participating. The atherogenic role of the NLRP3 inflammatory trigger in HIV infection has not been studied in detail. Awareness of the mechanism of the inflammatory complex and its involvement in HIV atherosclerosis can help explain the current clinical trials aimed at this pathway [65].

### 3.4. Autophagy Inhibition

Autophagy is programmed and regulates the clearance of aggregated toxic proteins and the degradation of damaged organelles by combining with lysosomes to aid cell viability. In response to pathogens (including HIV), autophagy reacts with the three previously mentioned molecular mechanisms. This autophagic effect also involves responses to inflammatory trigger signaling pathways that lower oxidative stress and ER stress [66].

Autophagy has three forms, which are classified by differences in (1) physiological properties and (2) methods of delivery to the lysosome. Yet, all three forms involve the same three stages. First, autophagy is activated, then the damaged protein or organelles are detached in the membrane-bound autophagosome, and then the autophagosome, binding to the lysosome, contributes to degeneration and recycling. Autophagy promotes plenty of disease progression, including inflammatory diseases, neurodegeneration, and cardiovascular disease. Studies of the importance of autophagy in inflammation support the anti-inflammatory role due to the specific capture and degeneration of triggered inflammatory complexes and cytokines. Autophagy basal levels range according to various cell types and can also be controlled by other signaling mechanisms [67,68].

In relation to atherosclerosis, autophagy is of great importance, since it protects cells from oxidative stress, lowering apoptosis and elevating the stability of plaques. Autophagic mechanisms also protect endothelial cells from oxLDL-induced cytotoxicity and help to avoid the development of foam cells in the vessel walls. In addition, autophagy is also a protection against endothelial cell apoptosis and SMCs. Due to the lack of autophagy, necrosis in atherosclerotic plaques grows high, elevating the sensitivity to instability and disruption in a mouse model of atherosclerosis with a deficiency of low-density lipoprotein (LDL) receptors (*LDLR*^−/−^) [43,69].

**Figure 1:**
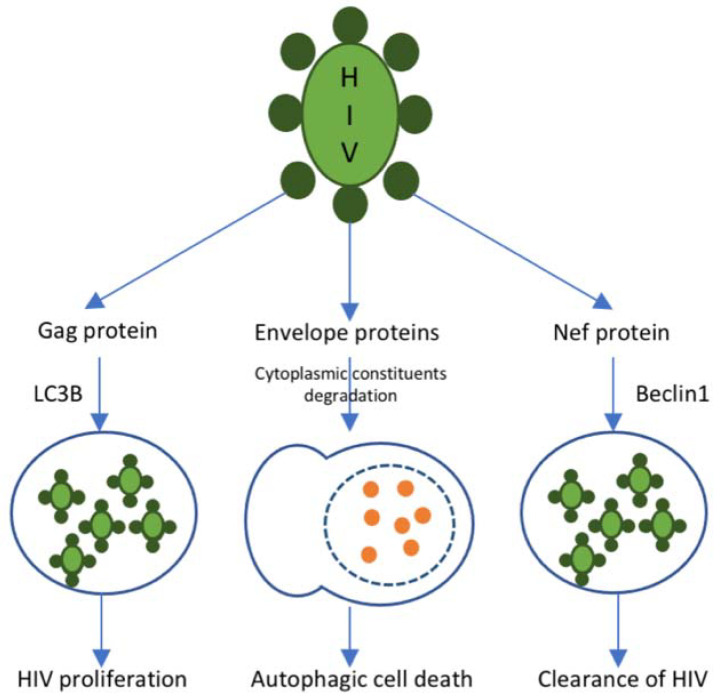
Autophagy in HIV. The autophagic response to HIV infection is regulated by different proteins presented on the surface of the HIV particle. Gag protein is presented in the HIV nucleocapsid. It mediates the sequestration of viral particles into autophagosomes through an interaction with the autophagy marker LC3B. Such autophagosomes do not fuse with lysosomes and contribute to viral proliferation. R5, X4, and other envelope proteins trigger autophagy-mediated host cell death. Nef protein interacts with Beclin-1 and, thus, suppresses the autophagy-mediated clearance of HIV.

Autophagy is the most essential catabolic process that promotes cell viability in response to various stressors. In the presence of HIV, it protects infected immune cells, which contributes to the decrease of elevated oxidative ER stress. The schematic representation of autophagy process in HIV issummarized in Figure 1. According to the cell type, the role of autophagy is able to range from assistance in T cell loss to a macrophage trigger. In dendritic cells, the trigger of autophagy aggravates the immune system response and even increases the infection of CD4^+^ T cells [70]. In macrophages, the induction of autophagy is elevated; nevertheless, the maturation of the cycle is distinctly suppressed, which may benefit from persistent immune activation. The autophagy molecular mechanism and its role in HIV-related atherosclerosis progression is able to range according to the types of cells involved. Suppression of autophagy in macrophages during HIV infection may be the main stimulus for HIV-associated atherosclerosis. The role of autophagy in HIV infection is well known, although its specific complicity in HIV-mediated atherosclerosis has not been widely studied [71].

Thus, there is strong evidence for all the three pathways in atherosclerosis and HIV infection: (1) oxidative stress, (2) ER stress, and (3) the formation of inflammatory cells and autophagy. However, the contribution that each of these molecular mechanisms makes in HIV-mediated atherosclerosis is now quite obscure. The combination of HIV infection, ART, and concomitant diseases causes oxidative stress, resulting in ER stress and increased UPR production, which, in turn, is able to result in the development of inflammatory diseases. HIV infection enables the inflammasome, mediating the release of inflammatory cytokines (e.g., IL-1β and IL-18), resulting in the atherosclerosis trigger and progression [38]. Subsequently, HIV suppresses autophagy, which increases the consequences of stress. Future analyses of these key molecular mechanisms should take into account the HIV-related fact that vascular inflammation may not be similar to HIV-mediated lymph node inflammation. Animal models are important for analyzing and evaluating the role of each of these mechanisms in HIV-related atherosclerosis, as well as the further effect of concomitant diseases or HIV-related treatment on these mechanisms [72].

## 4. Managing Atherosclerosis in HIV

The initiation of HIV infection is accompanied by reduction in total cholesterol, LDL-C, and HDL-C levels. In a study comparing patients with HIV infection with the corresponding non-infected control groups, the group with HIV carriers had decreased levels of HDL and LDL cholesterol and increased levels of triglycerides, CRP, and IL-6126. Since it is currently recommended to start ART during the initial diagnosis of HIV, the lipid picture of untreated HIV infection is only observed in people who are in conditions of limited resources and, therefore, are unable to obtain and start ART. It is noteworthy that in a meta-analysis of eighty studies, it was recorded that a high possibility of CVD events in HIV infection occurs in sub-Saharan Africa and the Asia-Pacific region [73].

Antiretroviral therapy’s influence on the levels of blood lipid ranges depending on the ART drug classes and also among drugs of the same class. The main data on effects of various drugs on lipid profile is summarized in Table 1.The effect of each individual drug is difficult to determine, since HIV therapy usually involves the use of a combination of several drugs. As a rule, protease inhibitors, NRTI, and non-NETI (NETI), elevate the level of triglycerides and are able to elevate the level of LDL-C. LDL-C and triglyceride levels elevate more with double therapy than with single therapy with protease inhibitors [74].

Significant distinctions were made between protease suppressors. Non-nucleoside reverse transcriptase inhibitor (NNRTI) also raises the levels of LDL cholesterol. However, it does not lower HDL cholesterol levels. Modern antiretroviral therapy, for example, integrase inhibitors, CC-chemokine receptor 5 (CCR5)-co-receptor antagonist (maraviroc), and second-generation protease inhibitors (such as atazanavir), have a beneficial influence on lipid levels and are also linked with enhanced surrogate markers of atherosclerosis, such as flow-mediated vasodilation and intima density of the carotid arteries. The integrase inhibitors dolutegravir and raltegravir apparently influence blood lipids in a comparable way [82,83].

Some studies show that the hyperlipidemia spread among people with HIV varies from 28% to 80%. It is noteworthy that hypertriglyceridemia is the most common anomaly. In 2018, in a meta-analysis of 14 studies involving 21,023 people from sub-Saharan Africa, antiretroviral therapy was linked with a high risk of hypertriglyceridemia (RR 2.05, 95% CI 1.51–2.77). Despite this fact, no consistent association was detected between antiretroviral therapy and high blood pressure, blood glucose, glycated hemoglobin (HbA1c), or other blood lipids [84].

One study showed that hypertriglyceridemia in patients with HIV infection was associated with an increased intake of total fat, saturated fat, and cholesterol in contrast to people without HIV infection. Saturated fat intake was greatly correlated with triglyceride levels. Therefore, there is a theory that changing the diet to reduce the intake of saturated fats could be a better approach to monitor and regulate raised triglyceride levels in HIV+ patients [85].

Until recent times, the leading principles for controlling cholesterol levels did not specifically concern people living with HIV. The main principles of the ESC/European Society for Atherosclerosis (EAS) 2016 highlighted a small section with information for HIV carriers that contains the following recommendations: change your diet, exercise, as well as switching, when possible, to a more “lipid-friendly” antiretroviral therapy [81]. In the same recommendations, it is noted that statin therapy should be kept in mind to achieve the target level of LDL-C 2.8 mmol/L, which is the same target level that is suggested for other patients with great risk of cardiovascular diseases. The National Lipid Association of the USA suggests that when choosing a drug treatment to reduce the level of LDL-C, HIV infection should be considered as a separate risk factor. The ACC/AHA guidelines of 2018 report that HIV infection may be considered as a factor that leads to a high risk of cardiovascular events, which would facilitate the initiation of moderate or high-intensity statin therapy [86,87].

Multiple clinical trials have proved that reducing the level of LDL cholesterol, usually due to the contribution of statin therapy, leads to a decrease in the risk of CCP in a large number of patients who are not carriers of HIV infection. At the same time, the 140-DELAY trial, which was launched in 2015, can be a solution to this complexity. In a small randomized study, it was observed that in patients with HIV infection, atorvastatin significantly lowered the volume of uncalcified coronary plaque in contrast to placebo during the follow-up1 year later [88].

When prescribing lipid-lowering drugs to HIV carriers, drug interactions are extremely important considerations. A holistic review of 18 statin studies in HIV carriers who received antiretroviral therapy demonstrated that statin therapy is able to be safely performed in this patient population. Treatment with lovastatin and simvastatin with protease suppressors is contraindicated, since there is a high risk of rhabdomyolysis regarding the high level of statins in the blood [89]. Apparently, treatment with ritonavir with protease suppressors also increases the area under the curve for atorvastatin; for this reason, the recommendations of the American Society of Infectious Diseases suggest that people receiving protease-inhibitor-based regimens should start with lower doses of atorvastatin. The blood levels of rosuvastatin elevate when it is used with atazanavir-ritonavir and lopinavir-ritonavir. Thus, for this reason, it is strongly recommended to limit the rosuvastatin dose to 10 mg while using a combination of these drugs [90].

Using pravastatin and fluvastatin is better in combination with antiretroviral therapy, but it should be kept in mind that they do not reduce the level of LDL cholesterol as much as atorvastatin or rosuvastatin. Previously, pravastatin and fluvastatin were popular to use after the introduction of antiretroviral therapy, but now this has changed, since it is known that higher degrees of reduction in LDL cholesterol lead to a greater decrease in the risk of CVD [91,92]. For some people with HIV infection, pitavastatin may be a good alternative. This drug is metabolized by glucuronidation, which prevents interactions between other drugs. The drug also shows a moderate decrease in the level of LDL cholesterol at higher doses. In one randomized study, it was reported that in HIV carriers, pitavastatin with a daily dose of 4 mg decreased the LDL cholesterol level by 31%, while pravastatin with a daily dose of 40 mg decreased the LDL cholesterol level by 21% compared to the baseline level, with equally low side effects for both statins [93,94]. A 2018 study reports that up to 50% of HIV carriers could qualify for statin therapy on the basis of at least one US guideline. However, not all of these people were prescribed statins. Even with statin therapy, HIV carriers may not achieve the same level of lipid reduction as non-HIV carriers. A meta-analysis of the study in HIV carriers showed that only the lower part reached the expected decrease in the level of LDL-C after starting statin therapy. During 2007–2014, the number of HIV carriers taking statins, which are off-limits due to their antiretroviral therapy, reduced. Despite this, the downward trend was weakened in 2015 due to an elevation in the use of cobicistat ART [95].

For HIV carriers not taking statins, ezetimibe is a good alternative (although with limited effectiveness in reducing LDL cholesterol levels). This drug is able to be used as an additional drug to the maximum tolerated dose of statins for HIV carriers who are at risk of cardiovascular diseases and also do not achieve a satisfactory reduction in LDL cholesterol levels with statin therapy alone [96]. The use of bile acid sequestrants in conditions of HIV infection is not very meaningful, for the reason that these agents increase the level of triglycerides in blood plasma, and their influence on the absorption of ART is unknown. Often, people with HIV infection may have a high load on the body. Therefore, additional therapy can create unnecessary problems [34].

Switching antiretroviral therapy to drugs that do not have a negative effect on blood lipids is important as long as the suppression of the virus persists. The transition from old protease suppressors to integrase suppressors improves the level of lipids in the blood, but due to an elevation in the frequency of virological insufficiency, and that is why it is not advised for people with a history of virological insufficiency [97]. In another study of HIV carriers who also have a high risk of cardiovascular diseases, continuation of the ritonavir-enhanced protease suppressor regimen or switching to dolutegravir (an integrase suppressor) was linked with comparable indicators of virological insufficiency after 48 weeks; nevertheless, the levels of total cholesterol, LDL-C, and triglycerides improved (*p* < 0.0001) in the dolutegravir group. For those who are not yet taking statins, adding statin therapy is more recommended than switching the type of ART. One study reports that the addition of rosuvastatin (10 mg per day) was better received and demonstrated better results on blood lipids than switching [98].

The main issue with hypertriglyceridemia in HIV carriers is very desirable to solve by reducing the consumption of carbohydrates, including alcohol. At the moment when the level of triglycerides in the plasma exceeds 10 mmol/L, there is a huge risk of pancreatitis, which, accordingly, requires immediate treatment. At the same time, lower levels of hypertriglyceridemia are not benign, since they are highly likely to elevate the risk of CVD. In people who are not carriers of HIV infection, fibrates are widely used to reduce triglyceride levels, although they have drug interactions with statins and some types of antiretroviral therapy. Switching to drugs that cause less hypertriglyceridemia is often the best approach [99].

## 5. Conclusions

The development of antiretroviral therapy is one of the most significant advances in modern medicine, which makes it possible to keep such a terrible disease as HIV under control. Unfortunately, in the issue of atherosclerosis, everything is not so rosy. Nevertheless, the available therapeutic techniques make it possible to alleviate the condition and slow down the development of pathology. Concerning atherosclerosis in HIV carriers, corrected techniques for slowing atherogenesis have also been described. Despite the leading role of immunity in both pathologies, the development of atherosclerosis in HIV carriers differs very little from atherogenesis in people without HIV, which once again confirms the importance and success of antiretroviral therapy. All observations are made on patients receiving this therapy in full, i.e., their viral load is almost undetectable.

## Figures and Tables

**Table 1:** Effects of the various ART drugs on the lipid profile.

Drug	Effect on Lipid Profile	Reference
Ritonavir (protease inhibitor)	HDL-C raised to 2.0 mmol/L; increased level of triglycerides in blood plasma; increased cholesterol level	[75,76,77]
Saquinavir (protease inhibitor)	Increased HDL cholesterol levels	[75]
Nelfinavir (protease inhibitor)	HDL-C raised to 1.2 mmol/L	[75,76]
Indinavir (protease inhibitor)	HDL-C raised to 0.8 mmol/L	[76]
Efavirenz (NNRTI)	Increased levels of total cholesterol, HDL cholesterol, LDL cholesterol, and triglycerides	[78,79]
Tenofovir alafenamide (NNRTI)	Increased LDL-C levels and HDL-C levels	[74,80,81]
Tenofovir disoproxil fumarate (NNRTI)	Hypolipidemic effect	[74,80,81]

## Data Availability

Not applicable.
